# Mitochondria-Associated Endoplasmic Reticulum Membranes Microenvironment: Targeting Autophagic and Apoptotic Pathways in Cancer Therapy

**DOI:** 10.3389/fonc.2015.00173

**Published:** 2015-07-27

**Authors:** Simone Patergnani, Sonia Missiroli, Saverio Marchi, Carlotta Giorgi

**Affiliations:** ^1^Laboratory for Technologies of Advanced Therapies (LTTA), Section of Pathology, Oncology and Experimental Biology, Department of Morphology, Surgery and Experimental Medicine, University of Ferrara, Ferrara, Italy

**Keywords:** MAM, autophagy, apoptosis, calcium, cancer, cell death

## Abstract

Autophagy is a tightly regulated catabolic pathway that terminates in the lysosomal compartment after the formation of a cytoplasmic vacuole that engulfs macromolecules and organelles. Notably, autophagy is associated with several human pathophysiological conditions, playing either a cytoprotective or cytopathic role. Many studies have investigated the role of autophagy in cancer. However, whether autophagy suppresses tumorigenesis or provides cancer cells with a rescue mechanism under unfavorable conditions remains unclear. Mitochondria-associated membranes (MAMs) are juxtaposed between the endoplasmic reticulum and mitochondria and have been identified as critical hubs in the regulation of apoptosis and tumor growth. One key function of MAMs is to provide asylum to a number of proteins with tumor suppressor and oncogenic properties. Accordingly, mechanistic studies during tumor progression suggest a strong involvement of these proteins at various steps of the autophagic process. This paper discusses the present state of our knowledge about the intimate molecular networks between MAMs and autophagy in cancer cells and addresses how these networks might be manipulated to improve anticancer therapeutics.

## Cell Death and Autophagy

Cell death is a fundamental biological process that is highly evolutionarily conserved and occurs in all multicellular organisms during the course of normal development and during adulthood. Programmed cell death (PCD) is required to maintain tissue homeostasis, to remove abnormal or damaged cells, and to remodel and sculpt tissues during morphogenesis (1, 2).

Perturbations to the normal patterns and regulation of cell death can contribute to several pathologies, such as neurodegenerative diseases, autoimmune diseases, and cancer. Not surprisingly, more than one form of cell death exists. Caspase-dependent apoptosis is considered the principal pathway in mammals. However, various additional forms of cell death have been described more recently and include necroptosis, pyroptosis, and autophagic cell death (3, 4).

Apoptosis and macroautophagy (hereafter, referred to as autophagy) play major roles in determining cellular fate and are discrete cellular processes that are mediated by distinct groups of regulatory and executioner molecules (4–6).

Apoptosis is the best-described PCD mechanism and is characterized by a series of morphological changes and plasma membrane blebbing, leading to the formation of apoptotic bodies.

The molecular biology of apoptosis has been studied over the last 40 years. The two most prominent molecular mechanisms that regulate the cell death process are the extrinsic and intrinsic pathways (7).

In the extrinsic pathway, the ligation of ligands (e.g., FasL, also known as CD95L), tumor necrosis factor-α (TNF-α), TNF-α-related apoptosis-inducing ligand (TRAIL, also known as APO2L), or TNF-α ligand superfamily member 10 (TNFSF10), with their respective death receptors, stimulate the recruitment of adaptor proteins, such as Fas-associated via death domain (FADD), which then recruits the initiator procaspase-8 or -10, causing their dimerization and activation (8).

Unlike the extrinsic pathway that regulates apoptosis through specialized death signals, the intrinsic pathway (also known as “mitochondrial apoptosis”) is mediated by several intracellular and extracellular signals, including endoplasmic reticulum (ER) stress, loss of survival/trophic factors, toxins, radiation, hypoxia, oxidative stress, ischemia–reperfusion injury, and DNA damage. As a result, these signals converge on mitochondria to induce mitochondrial outer membrane permeabilization (MOMP), which contributes to multiple redundant lethal events, including caspase cascades, the activation of caspase-independent pathways for cellular dismantling and bioenergetic failure (9, 10) via mitochondrial permeability transition pore opening (11). In particular, MOMP proapoptotic factors (apoptogens) that are normally found in the intermembrane space (IMS) are irreversibly released into the cytoplasm. A critical apoptogen that is released from the intermembrane mitochondria space is cytochrome c (Cyt c). In the cytosol, Cyt c induces the apoptosis protease-activating factor 1 (APAF-1) and ATP/dATP to assemble the apoptosome, a molecular platform that promotes the proteolytic maturation of caspase-9. Finally, caspase-9 recruits and activates effector caspases.

Autophagy is a lysosome-mediated, multistep self-degradation process and is distinct from other degradative pathways such as proteasomal degradation, in which components are degraded to supply energy (12, 13). During autophagy, an isolation membrane sequesters a small portion of the cytoplasm, including cytosolic materials and organelles, to form the autophagosome. Autophagosomes fuse with lysosomes to yield autolysosomes, which degrade internalized materials.

This process is executed and regulated by a large group of distinct autophagy-related (Atg) proteins, which direct the four major steps of the autophagic machinery: initiation, nucleation, cycling, and expansion/closure. More than 30 Atg genes are known. Readers interested in the specialized role of Atg proteins should consult recent reviews (14–16). The cofactors involved in autophagosome processing are described as follows. Upon induction of autophagy, the main negative regulator of the catabolic process, MTOR (mechanistic target of rapamycin), is suppressed and inactivated. This event activates the ULK1 (unc-51 like kinase 1)-complex (including ULK1, Atg13, FIP200, and Atg101), resulting in the ULK1-dependent phosphorylation of Atg13, FIP200, and ULK1 itself and translocation of the complex to the ER, the major site of autophagosome formation (17). At these activation levels, ULK1 also regulates the activity of the class III phosphatidylinositol (PtdIns) 3-kinase complex (including Beclin1, Atg14(L)/barkor, Vps15, Vps34, and Ambra1). This complex generates PI3P, which interacts with DFCP1, Atg2, and WIPI1, and recruits other Atg proteins involved in membrane elongation. Additionally, other complexes are fundamental for the autophagosome elongation, such as two ubiquitin-like proteins. Atg7 and Atg10 mediate the association of the Atg5–Atg12 complex with Atg16L1. The Atg12–Atg5–Atg16L1 complex and the LC3 (Atg8 homolog)–phosphatidylethanolamine (PE) conjugate play important roles in the elongation and closure of the isolation membrane. The Atg12–Atg5–Atg16L1 complex is also required for the formation of a covalent bond between LC3 and PE. When autophagosome formation is concluded, the outer autophagosomal membrane fuses with lysosomes, releasing autophagic cargo into the lysosomal lumen (18).

Basal levels of autophagy are important for maintaining cellular homeostasis. The physiological importance of autophagy in maintaining tissue homeostasis has been demonstrated in different organs, including brain, liver, heart, muscle, kidney, and adipose tissue (12, 19, 20). Nutrient deprivation is the main activator for autophagy induction, but is not the only mechanism. For example, decreased levels of specific amino acids and increased levels of glucocorticosteroids and thyroid hormones also stimulate the catabolic process (21). Furthermore, it is clear that autophagy is an important energy generator. Indeed, amino acids obtained by autophagy can be converted into intermediates of the tricarboxylic acid (TCA) cycle and contribute to ATP production. The breakdown of lipid droplets by autophagy (i.e., lipophagy) may also account for this energy-producing role, especially in the liver (22). At molecular levels, nutrient failure modulates the activity of important energy-sensing proteins. One of these proteins is AMPK (5′ AMP-activated protein kinase), a conserved sensor of intracellular adenosine nucleotide levels. AMPK is activated when levels of AMP or ADP increase in response to slight decreases in ATP production. This kinase is significantly involved in autophagic regulation via mechanisms wherein MTOR activity is suppressed. Furthermore, MTOR regulates mitochondrial ATP production and determines the health of the mitochondrial population by a process termed mitophagy. Previous studies have shown that these events are also regulated by AMPK-dependent MTOR inactivation (23).

In addition, a function for autophagy in cell death has long been proposed. Cell death is often attributed with high levels of autophagosomes and active autophagy, and hence, the term “autophagic cell death” is often used.

Many publications have reported that death stimuli in mammalian cells and non-mammalian systems are caused by autophagy (24). However, it is unclear whether autophagy occurs prior to apoptosis or during the dismantling of cellular mechanisms (25).

Furthermore, autophagy has been implicated in cell death for various pathological conditions, such as neurodegeneration, immunity, and aging and especially in cancer (26).

## Tumor-Suppressing and Tumor-Promoting Roles of Autophagy in Cancer

A number of recent studies have revealed that autophagy can function as either a pro-death or pro-survival mechanism in cancer cells. Depending on the different stages of tumor development and the cell type, autophagy acts in two opposing capacities. During early stages of cancer, autophagy has preventive effects; however, after sufficient tumor development, cancer cells utilize autophagy mechanisms to provide energy for sustained growth (Table 1).

**Table 1:** **Functions of autophagy in cancer**.

Autophagy in tumor promotion	Autophagy in tumor suppression
Tumor cells use autophagy to adapt in a hypoxic environment	Contributes to cancer cells death at early stage of several cancer types
Autophagy is activated as a protective mechanism to mediate the acquired resistance phenotype of some cancer cells during chemotherapy	Autophagy suppresses tumor initiation limiting the accumulation of damaged proteins and organelles such as mitochondria and peroxisomes
Autophagy as a mechanism that permits obtaining both ATP and metabolic intermediates	Autophagy may protect against tumor initiation and development by favoring cellular differentiation, increasing protein catabolism, or promoting autophagic cell death

The role of autophagy as a potent tumor suppressor mechanism was inferred from the observation that its molecular pathways were frequently ablated in tumor cells.

During tumor initiation, autophagy is a protective process that limits the accumulation of harmful proteins and organelles through its intrinsic quality control activities. These tumor suppressor properties are primarily attributed to protection against genotoxic stress, such as the elimination of reactive oxygen species (ROS) (27). Autophagy-mediated ROS removal inhibits the deleterious effect of ROS on DNA mutations that have been extensively shown to induce tumorigenesis (28). Autophagy primarily prevents ROS accumulation through the elimination of damaged mitochondria via a selective form of autophagy (i.e., mitophagy) (29). The tumor-suppressing roles of autophagy are also related to the regulation of the inflammatory process, which has been compared with the initiation of cancer (30–32).

The role of autophagy in tumor suppression can also be observed during the upregulation of members of the phosphatidylinositol 3-kinase (PI3K) family to similar levels as the MTOR and AKT kinases (33, 34). These positive regulators of cell growth inhibit PTEN (phosphatase and tensin homolog deleted on chromosome 10) activity and other tumor suppressors, such as LKB1 (liver kinase B1). These proteins are deregulated in cancer cells and are potent activators of the autophagic machinery (35).

Furthermore, AKT inhibits autophagy in an MTOR-independent manner via the phosphorylation of the essential autophagy and tumor suppressor protein BECLIN-1 (36). A BECLIN-1 mutation has been shown to confer resistance to AKT-dependent phosphorylation, increased autophagy, and inhibited AKT-driven tumorigenesis (36). As for AKT, the implications in cancer of the oncoprotein BCL (B-cell lymphoma)-2 and its antiapoptotic homologs (e.g., BCL-XL and MCL-1) are attributed to their regulatory roles in cell death and the autophagic process. In particular, antiapoptotic BCL-2 family members have been shown to suppress autophagy through their interaction with or inhibition by BECLIN-1 (37, 38). Thus, the inhibition of autophagy contributes to the transformation of a normal cell into a cancerous cell and favors a state of genomic instability, i.e., the ideal trigger for tumor initiation.

Another protagonist of this signaling pathway involving BCL-2 family members during autophagy is the putative tumor suppressor AMBRA1 (autophagy/beclin-1 regulator 1), which is a crucial factor in regulating autophagy in vertebrates. AMBRA-1 enhances the activity of BECLIN-1, and thus mediates autophagosome nucleation (39, 40).

BECLIN-1-mediated autophagy is negatively regulated through a direct interaction between BECLIN-1 and ER-BCL-2. Past studies have shown that the AMBRA-1-BECLIN-1 complex re-localizes to the ER upon autophagy induction and that AMBRA-1 is able to bind to the antiapoptotic factor BCL-2 (41).

A further potential molecular link between defective autophagy and tumorigenesis involves the autophagic-dependent protein p62/SQSTM1, responsible for removing damaged organelles and misfolded proteins that may cause DNA damage and genomic instability. p62/SQSTM1^−/−^ mice have been shown to be protected from RAS-induced lung carcinomas.

The predominant role of autophagy in tumor cells is to confer stress resistance and thereby maintain cancer cell survival. Indeed, tumor cells may use autophagy to increase the supply of limited nutrients. In addition, autophagy has been induced in hypoxic tumor cells distal from blood vessels, and cancer cells have been shown to upregulate the autophagic process to prevent metabolic stress induced by chemical and radiological therapies. Based on these studies, there are a number of potential anticancer approaches targeting the autophagic process. As such, autophagy-modulating agents have been studied in preclinical models (Table 2) (42).

**Table 2:** **MAM cofactors regulating autophagic machinery**.

Protein	Regulation of autophagy	Potential molecular targets
AKT	Negative	BECLIN-1/MTOR
BCL-2	Negative	BECLIN-1/AMBRA-1
BCL-XL	Negative	BECLIN-1
AMBRA-1	Negative	BCL-2 family members
PML	Unknown	Regulate autophagy via ER-mitochondria cross-talk (hypothetical)
PKCβ/p66^Shc^	Negative	PKCβ-dependent mitochondrial translocation of p66^Shc^
p53	Negative	Regulate autophagy via ER-mitochondria cross-talk (hypothetical)
MFN-1/MFN-2	Positive	PINK1/Parkin-dependent mitophagy
DRP1	Positive	PINK1/Parkin-dependent mitophagy
HRAS	Dual role	Class I PI3K/AKT/MTOR pathway (negative), Rac1/MKK7/JNK pathway (positive)
MTOR	Negative	Class I PI3K, AKT, PINK1
PP2A	Positive	MTOR, PML (hypothetical)

In particular, the most promising anticancer agents are autophagy inhibitors (such as chloroquine, CQ, and its derivative hydroxychloroquine, HCQ), which are able to enhance cell cytotoxicity when combined with different anticancer drugs (Table 3) (43). Interestingly, several studies have shown how rapamycin and its analogs, i.e., allosteric MTOR inhibitors, in combination with autophagy inhibitors (e.g., HCQ, bafilomycin A1, and methyladenine) increased *in vitro* and *in vivo* cytotoxicity of human cancers (44, 45). The efficacy of this combination may be explained as follows. The aberrant expressions of PI3K/AKT/MTOR are found in several tumor types and are important to the initiation and progression of cancers. These cofactors are critical negative regulators of the autophagic machinery. When their activity or expression is blocked, autophagy may be induced and may trigger consequent autophagic-related resistance that promotes cancer cell survival. The resistance of cancer cells to AKT/MTOR inhibitors is a significant issue in a number of different tumor cells, such as relapsed mantle cell lymphomas. Pretreatments with autophagic inhibitors can effectively overcome this resistance by inhibiting AKT/MTOR activity, blocking the autophagic process, and activating the apoptotic pathway. The targeting of the PI3K/AKT/MTOR autophagy pathways can overcome cancer cell resistance to chemotherapy and radiotherapy. However, the CQ and HCQ studies should be interpreted carefully because the reported effects may be mediated by mechanisms unrelated to autophagy inhibition. For example, a recent study showed that a CQ treatment reduced hypoxia and cancer cell metastasis and improved chemotherapy efficacy rates and responses in an autophagic-independent manner (46).

**Table 3:** **Some of the clinical trials combining the autophagy inhibitor HCQ**.

Cancer type	Drugs combination	Phase trial
Breast cancer	HCQ + lixabepilone	I/II
Pancreatic cancer	HCQ + gemcitabine	I/II
Pancreatic cancer	HCQ + capecitabine + photon radiation	II
Glioblastoma	HCQ + temozolomide	I/II
Non-small-cell lung cancer	HCQ + cisplatin etoposide	I/II
Non-small-cell lung cancer	HCQ + paclitaxel and carboplatin	II
Renal cell carcinoma	HCQ + high dose interleukin-2 and other systemic therapies	I
Metastatic colorectal cancer	HCQ + capecitabine, oxaliplatin, and bevacizumab	II
Colorectal cancer	HCQ + FOLFOX/bevacizumab	I/II
Ovarian cancer	HCQ + sorafenib	I
Multiple myeloma	HCQ + bortezomib	I/II
Chronic myeloid leukemia	HCQ + imatinib	II

Recently, various cancer and autophagy-related factors, including the aforementioned proteins, have been shown to localize at mitochondria-associated ER membranes (MAMs), which are the primary intracellular platforms that detect extracellular inputs and stressful conditions (47, 48). We next discuss the roles of oncogenes and oncosuppressors at MAMs in autophagy and consider the structural functions of the ER-mitochondria interface in the regulation of autophagy (49).

## MAM Structure

**Figure 1:**
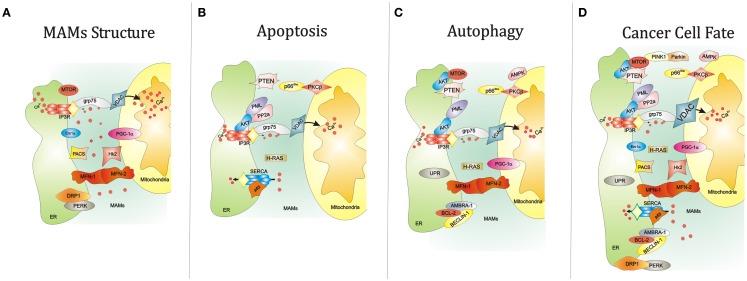
**Summary of the key functions of the ER-MAMs resident proteins**. **(A)** Several proteins reside in MAMs compartment and regulate the juxtaposition between ER and mitochondria, including grp75, Ero1α, PERK, and MFN-1/2. At the same time, other MAM resident proteins control the cell survival by governing apoptosis **(B)** and autophagy **(C)**. For example, it has been reported that PML, IP3R3, and AKT mutually interact to allow the correct Ca^2+^-movement between ER and mitochondria, an essential proapoptotic signal. Interestingly, the maintenance of this interorganelle Ca^2+^-communication is also important for the autophagic process. Of relevance, reductions of mitochondrial Ca^2+^ accumulation may trigger autophagy. In parallel, most of MAM proteins also govern the autophagic machinery. Of relevance, AKT and MTOR regulate negatively autophagic process. The same work is executed by the molecular axis composed by p66^Shc^ and PKCβ. It is widely accepted that autophagy is strictly linked to several human diseases, in particular cancer. Think about a possible link between autophagy, cancer, and MAMs is not so difficult. Several findings may suggest this. For example, the corrected maintenance of MAMs integrity by MFN-1/-2 and DRP1 is also crucial for autophagosome formation and tumor cell growth. **(D)** The autophagic regulator AMBRA-1 has been found to interact with both the oncogene BCL-2 and the tumor suppressor BECLIN-1. Again, ER stress mediated by UPR a potent autophagic activator: at the same time ER stress is a critical signal capable to drive cell death. Also, the activity of the main proteins involved in Ca^2+^ release and reuptake at MAM levels, SERCA and IP3R3, is reported to be involved in apoptosis and tumorigenesis. Interestingly, the functioning of these channels is intimately regulated by several oncogenes (like AKT) and tumor suppressor (such as p53), which are also involved in regulation of autophagy. Abbreviations: grp75, glucose-regulated protein 75; IP3R3, inositol 1,4,5-trisphosphate receptor type 3; MFN-1/-2, mitofusin-1/-2; Hk2, Hexokinase 2; PACS, phosphofurin acidic cluster sorting protein; VDAC, voltage-dependent anion-selective channel; Ca^2+^, calcium; PGC-1α, peroxisome proliferator-activated receptor gamma coactivator 1 alpha; PTEN, phosphatase and tensin homolog; p66^Shc^, 66 kDa proto-oncogene Src homologous-collagen homolog; PKCβ, protein kinase C beta; PP2a, protein phosphatase 2; AKT, protein kinase B; SERCA, sarco/endoplasmic reticulum Ca^2+^ ATPase; MTOR, mechanistic target of rapamycin; PML, promyelocytic leukemia; UPR, unfolded protein response; PINK1, PTEN-induced putative kinase 1; PERK, protein kinase-like ER kinase; DRP1, dynamin-related protein; Ero1a, ER oxidoreductase 1 alpha; IP3R3, inositol 1,4,5-trisphosphate receptor type 3; AMBRA-1, Beclin1-regulated autophagy; BCL-2, B-cell lymphoma 2; BECLIN-1, BCL-2-interacting protein;, AMPK, 5′ adenosine monophosphate-activated protein kinase).

MAMs are ER membranes that are similar to mitochondria. However, MAMs are not simply static bridges between the ER and mitochondria. On the contrary, interorganelle communication between the ER and mitochondria is crucial for processes such as lipid synthesis and transport, mitochondrial functions, the regulation of calcium homeostasis, and apoptosis (48). A large number of ER and mitochondria-associated proteins have been identified in MAMs, demonstrating the crucial involvement of MAMs in all physiological processes (Figure 1A). These proteins include various chaperones [e.g., glucose-regulated protein 75 (Grp75)], enzymes involved in ER redox regulation [e.g., ER oxidoreductase 1 alpha (Ero1α)] and protein kinases [e.g., ER stress sensor double-stranded RNA-activated protein kinase (PKR)-like ER kinase (PERK)]. Other proteins are also responsible for regulating mitochondrial dynamics and morphology, such as the mitofusins MFN-1 and MFN-2 and the dynamin-like protein DRP1 (50).

The ER is also the major site of Ca^2+^ storage in mammalian cells. The movement of Ca^2+^ between the ER and mitochondria is an essential component of the cell survival processes.

Several studies have examined the distribution of Ca^2+^ between the ER and mitochondria (51–53). A study by Csordas and colleagues provided direct evidence for the existence of high-Ca^2+^ microdomains at ER-mitochondria contact sites that are regulated by the area and gap width of ER-mitochondria interconnections. Furthermore, they described a novel approach for determining the [Ca^2+^]_ER-mt_ in microdomains (54).

Based on these studies, MAMs regulate several Ca^2+^-dependent cellular processes. The movement of Ca^2+^ between the ER and mitochondria is essential for the correct execution of both apoptotic (55) and autophagy pathways (56). Several Ca^2+^ channels regulating the apoptotic program are located in the MAM compartment. For example, IP3R3 (located at the ER) and VDAC (situated at the outer mitochondrial membrane) are highly concentrated in MAMs (57).

Furthermore, growing bodies of evidence shows that the ER-mitochondria contact sites contribute to autophagosome formation and that proteins in the MAM compartments are indispensable for proper autophagic vesicle formation (49, 58–60).

## MAMs, Novel Regulators of Cancer Cell Fate?

Although the ER–mitochondria interface is known to assist the autophagic pathway, the presumed link between the regulation of the autophagy/MAMs axis and cancer progression has not been addressed.

The role of MAMs in the regulation of apoptotic cell death in cancer is partially understood. In particular, several proteins are known to exert their pro- and antiapoptotic functions between the ER and mitochondria (57) (Figure 1B).

Furthermore, most of these proteins possess tumor suppressor and oncogenic properties and are mutated or deleted in various types of human cancers.

One classical example is the tumor suppressor PML (promyelocytic leukemia protein). This protein performs its proapoptotic functions through the assemblage of nuclear macromolecular structures called PML nuclear bodies (PML-NBs) (61). A recent study found that this protein localizes to the ER and MAMs, where it regulates Ca^2+^-dependent apoptosis by blocking the activity of IP3R3 (62). PML is one component of a complex composed of PP2A (protein phosphatase 2), IP3R3 (inositol 1,4,5-trisphosphate receptor, type 3), and AKT. Several studies have demonstrated that AKT mediates the phosphorylation of IP3R3 with a consequent decrease in Ca^2+^ transfer from the ER to the mitochondria (63–65).

The significant accumulation of mitochondrial Ca^2+^ amplifies the apoptotic signal. Key proteins (such as IP3R3, VDAC, and the permeability transition pore-complex) have also been demonstrated to be critical components for the ER-triggered, proapoptotic mitochondrial membrane permeabilization process (66).

Other tumor suppressors have also been observed to localize in the MAM compartment and regulate Ca^2+^ flux and apoptosis by regulating AKT activity.

In fact, PTEN was recently shown to interact with the AKT/IP3R complex, leading to a reduction in its phosphorylation and an increase in Ca^2+^ release (67).

In addition, the most studied tumor suppressor (p53) regulates tumorigenesis via a Ca^2+^-dependent pathway. A recent study found that p53 localizes to the ER and MAMs, where it modulates the ER-mitochondria cross-talk and the Ca^2+^ transfer from the ER to the mitochondria. As a result, mitochondria accumulate significant amounts of Ca^2+^, leading to an alteration in the morphology of this organelle and the induction of apoptosis (68, 69).

Other indications of the close relationship between MAMs and cell death can be found in the link between the SHC1 gene (encoding for the p66^Shc^ protein) and the putative oncogene PRKCB (encoding for the protein kinase C, beta, PKCβ). p66^Shc^ is a MAM protein (70) that is regulated by ROS and involved in the regulation of Ca^2+^ homeostasis (71, 72). Once activated, p66^Shc^ translocates into mitochondria where it is reported to influence the cell life span and apoptosis through the perturbation of mitochondrial functions. PKCβ is required for the phosphorylation and activation of p66^Shc^ at Ser36 and for its translocation into the mitochondrial compartment (72).

One of the most mutated oncogenes in human cancer, H-RAS, is localized in MAMs, where it exerts its antiapoptotic function by modulating Ca^2+^-dependent apoptosis. Notably, the activation of oncogenic H-RAS leads to a perturbation in Ca^2+^-handling and dysfunction in mitochondrial physiology (73).

Intriguingly, these proteins are also known to regulate the autophagic pathway. Thus, the localization of these players in the MAM compartment could be important for the regulation of autophagy (Figure 1C).

Okadaic acid (OA) is a PP2A-blocking drug. Several works have identified a direct role for this compound in the regulation of autophagy (74–76).

Interestingly, PML maintains an appropriate Ca^2+^ flux in the MAM compartment by mediating the recruitment of PP2A (62). Given that treatment with OA interferes with this molecular pathway, PML may regulate autophagy via ER-mitochondria cross-talk. The same issue is relevant to AKT. Several class I PI3K inhibitors (such as LY294002 and wortmannin) negatively regulate autophagy through AKT inhibition (77). Preclinical studies have also suggested the use of these compounds in cancer treatment (78). The main inhibitor of the MTOR kinase, rapamycin, was found to regulate AKT, and its use has been approved in several clinical trials. Considering the important role of regulating AKT activity to modulate cell death during cancer, the specific and direct roles of MAM compartment-localized AKT needs to be addressed (78). In addition, AKT may be directly phosphorylated and activated by MTOR.

MTOR also modulates the cellular distribution of mitochondria (79, 80) and the fate of this organelle when associated with the MTOR complex 2 (MTORC2) (81). This complex is activated by PINK1 (PTEN-induced kinase 1) (82), the main regulator of selective mitophagy (83). Interestingly, MTORC2 is also physically associated with MAMs, where it interacts with and phosphorylates AKT. Once activated, MTORC2-AKT signaling controls MAM integrity, mitochondrial metabolism, and cell survival by regulating the activity of MAM resident proteins PACS2, IP3R, and HK2 (84).

Thus, these findings suggest a mutual regulation between the MTORC2-AKT complex and the integrity and functionality of MAMs. Moreover, the pharmacological modulation of this MAM-localized complex could be used as a novel therapeutic intervention to modulate cell fate in cancers with AKT/MTOR mutations.

As reported earlier, the protein AMBRA-1 seems to be a fundamental regulator during autophagy induction, as evidenced by its interactions with BCL-2 family members at the ER and mitochondrial compartments.

Interestingly, several molecular and pharmacological agents capable of interfering with BCL-2 activity (such as BH3-mimetic compounds) are already used in cancer therapies (85). Thus, these drugs may also modulate AMBRA-1 activities at the ER–mitochondrial interfaces.

Several works report that a few of these chemotherapeutic drugs modulate autophagy by regulating BCL-2 family member activities (86), which shows a possible and crucial involvement of AMBRA-1 protein activity in cancer therapies.

A growing body of evidence suggests the antidiabetic drug metformin as a novel candidate for cancer therapy (87, 88). However, the mechanism of action remains unknown. This compound drives mitochondrial bioenergetics, models the ER-mitochondria contact site, and acts as a selective inhibitor of PKCβ (89, 90). Because the role of p66^Shc^-PKCβ in the regulation of autophagy has been recently shown (91) and metformin can be used to manipulate the autophagic process (35, 92), metformin may also directly affect the p66^Shc^-PKCβ-mediated regulation of the cell life span at MAM sites.

A broad range of chemotherapeutic agents has been used to improve or restore p53 activity (93). Given the autophagic-modulating properties of this tumor suppressor and considering its novel apoptotic function in the ER-MAM compartment, a novel therapy based on the simultaneous modulation of the autophagic mechanism and the p53-dependent transfer of Ca^2+^ from the ER to mitochondria appears to be possible.

However, proteins with tumor suppressor and oncogenic properties are not the only proteins that have been linked to MAMs and cancer progression. As reported earlier, a large number of MAM proteins are required to maintain the proper communication channels between the ER and mitochondria. Most of these proteins play pivotal roles during the autophagic process.

Notably, the correct assembly of MAMs is also guaranteed by fusion and fission events in the mitochondrial compartment.

Mitochondrial fusion is primarily orchestrated by mitofusins-1 and -2 (MFN-1/-2) and DRP1 (94). Remarkably, these proteins are crucial for tethering the ER to the mitochondria and stabilizing MAMs. Fusion proteins are critical elements for the transfer of outer mitochondrial membrane proteins to autophagosomes (49). The proteins are also indispensable elements for the execution of PINK1/Parkin-dependent mitophagy (95, 96). Importantly, the modulation of MFNs and DRP1 activity has been proposed to prevent tumor cell growth in several human cancers (97). Mitochondrial biogenesis is a critical aspect of the integrity of MAMs. Over the past few years, transcriptional coactivators of the peroxisome proliferator-activated receptor gamma coactivator-1 (PGC-1) family have been shown to directly mediate the transcription of mitochondrial genes responsible for the biogenesis of the organelle. This family includes PGC-1α and PGC-1β, which are present in many cell types, including cells found in the heart, skeletal muscles, and brain (98). Interestingly, both isoforms were significantly upregulated in the chemo-resistant cells. In particular, the silencing of PGC-1β isoform has been shown to reestablish the sensitivity of cancer cells carrying mtDNA mutations to chemotherapy agents (99). However, activity of the alpha isoform has been linked to autophagic modulation (100). Thus, the action of PGC-1α may be modulated by several pharmacological compounds (101), many of which also regulate the autophagic machinery (such as AMPK activators). In addition, several works have reported that the disruption of PGC-1α expression could be a critical aspect of cancer progression (102).

A functioning and healthy ER is a critical component of the MAM integrity. When this aspect is lacking, ER stress increases and results in unfolded protein response (UPR), ER-associated degradation (ERAD), MAM structure alteration, and Ca^2+^ release. For prolonged or extreme stresses, these signaling pathways can lead to cell death (103). Accumulating data indicate that ER stress is also a potent trigger of autophagy. Depending on the context, autophagy counterbalances ER stress-induced ER expansion enhances cell survival or commits the cell to non-apoptotic death. In a cancerous environment, this subject is a matter of debate. Previous reports have indicated that the inhibition of autophagy in colon epithelial cells prevents cell death induced by an ER-MAM stressor (e.g., Ca^2+^, thapsigargin, and tunicamycin) (104). However, in other human cancers, certain ER-MAM-disrupting treatments (e.g., photodynamic therapy and vitamin D analog EB1089) may kill cancer cells by a mechanism that depends on autophagy (105–107).

**Figure 2:**
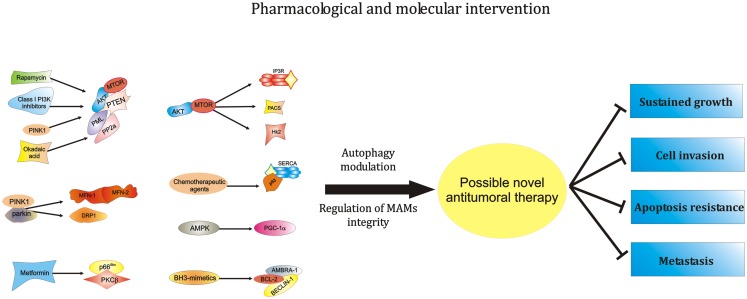
**Modulation of the activity of ER-MAMs protein may represent a novel therapeutical strategy against carcinogenesis**. MTOR, the main regulator of autophagic process, was found to modulate MAMs structure and activities. In addition, other PI3K family members, like AKT and PTEN, are located at ER-MAMs sites and represent critical regulator of autophagy and MTOR activity. Of relevance, these PI3K family members may be modulated by rapamycin and class I PI3K inhibitors. Furthermore, since the interaction of AKT-PTEN axis with the tumor suppressor PML and the protein PP2A at MAMs level is well described, it is possible to hypothesize a pharmacological intervention at this level to modulate the autophagic process during tumorigenesis. Also, the most studied tumor suppressor, p53, recently found to be located at ER-MAMs sites, was found to regulate autophagy. It is so clear that chemotherapic agents activating p53 may exert their antitumor activities at ER-mitochondria contact sites in an autophagy-dependent manner. Another critical regulator of autophagy, AMPK, was recently found to destabilize the correct juxtaposition between ER and mitochondria. Interestingly, the pharmacological AMPK-activator Metformin is found to regulate the activity of PKCβ. It is easy to think that metformin may interfere with the recent discovered negative modulator of autophagy, the p66^Shc^–PKCβ axis. Likewise, the potent autophagic regulator AMBRA-1 is found to be a potent controller of BCL-2 family member activities. Interestingly, the employment of BH3-mimetics is often used in chemotherapy, suggesting a possible role of AMBRA-1 during therapeutical approaches against cancer. Finally, the most important proteins involved in the mitophagic process, PINK1 and Parkin interact and regulate the activity of different ER-MAMs resident protein, thus suggesting the possibility of intercede to the activity of PINK-1-Parkin to develop novel antitumor approaches. Abbreviations: MFN-1/-2, mitofusin-1/-2; Hk2, hexokinase 2; PACS, phosphofurin acidic cluster sorting protein; PGC-1α, peroxisome proliferator-activated receptor gamma coactivator 1 alpha; PTEN, phosphatase and tensin homolog; p66^Shc^, 66 kDa proto-oncogene Src homologous-collagen homolog; PKCβ, protein kinase C beta; PP2a, protein phosphatase 2; AKT, protein kinase B; SERCA, sarco/endoplasmic reticulum Ca^2+^ ATPase; MTOR, mechanistic target of rapamycin; PML, promyelocytic leukemia; PINK1, PTEN-induced putative kinase 1; DRP1, dynamin-related protein; AMPK, 5′ adenosine monophosphate-activated protein kinase; PI3K, phosphoinositide-3-kinase; AMBRA-1, Beclin1-regulated autophagy; BCL-2, B-cell lymphoma 2.

Overall, these findings suggest that the correct maintenance of the ER-mitochondria interface is a critical part of the autophagic process. Because catabolic process and several proteins linked to MAMs are deeply involved in cancer progression, a number of novel therapeutic approaches based on the manipulation of MAMs can be proposed (Figures 1D and Figure 2).

## Concluding Remarks

Half a century ago, Christian de Duve coined the term “autophagy” to describe a process in which the cell digests its cytoplasmic materials via lysosomal degradation.

More than 20 years ago, Jean Vance biochemically isolated an intracellular structure representing the physical contact between the ER and mitochondria and termed this membrane fraction MAMs.

In the last decade, dysregulated autophagy has been associated with various types of disease-like phenotypes, including cancer.

In recent years, it has been revealed that the function and behavior of MAMs are central to the main molecular pathways of human cells, and consequently, MAM dysfunction has been associated with several types of cancer. In addition, a growing body of evidence suggests a key role for MAMs in the regulation of autophagy.

Together, these findings suggest that the MAM environment could be a fundamental background for the regulation of autophagic-dependent cancers. However, this intricate network has not been well addressed.

Future work will be required to better understand how this finely tuned compartment participates in various stages of autophagy during cancer development. These studies will help facilitate the rational design of combinatorial strategies aimed at modulating autophagy or MAM structures and resident proteins to elicit maximum therapeutic benefits against various types of human cancers.

## Conflict of Interest Statement

The authors declare that the research was conducted in the absence of any commercial or financial relationships that could be construed as a potential conflict of interest.
